# It is time to mobilize suicide prevention for sexual and gender minorities in Canada

**DOI:** 10.17269/s41997-020-00316-3

**Published:** 2020-04-23

**Authors:** Olivier Ferlatte, Travis Salway, John L. Oliffe, Elizabeth M. Saewyc, Cindy Holmes, Lynette Schick, Aaron Purdie, Diana (Dammy) Damstrom-Albach, Edward R.G. Mantler, Darren Ho, Rod Knight

**Affiliations:** 1 Department of Social and Preventive Medicine, School of Public Health, University of Montreal, Montreal, Canada; 2 Centre de Recherche en Santé Publique, Montreal, Canada; 3 Faculty of Health Sciences, Simon Fraser University, Burnaby, Canada; 4 British Columbia Centre for Disease Control, Vancouver, Canada; 5 Centre for Gender and Sexual Health Equity, Vancouver, Canada; 6 School of Nursing, University of British Columbia, Vancouver, Canada; 7 Department of Nursing, University of Melbourne, Melbourne, Victoria Australia; 8 Stigma and Resilience Among Vulnerable Youth Centre (SARAVYC), Vancouver, Canada; 9 McCreary Centre Society, Vancouver, Canada; 10 School of Social Work, University of Victoria, Victoria, Canada; 11 Mental Health Commission of Canada, Ottawa, Canada; 12 Health Initiative for Men, Vancouver, Canada; 13 Canadian Mental Health Association, BC Division, Vancouver, Canada; 14 Community-Based Research Centre, Vancouver, Canada; 15 British Columbia Centre on Substance Use, Vancouver, Canada; 16 Department of Medicine, University of British Columbia, Vancouver, Canada

**Keywords:** Suicide, Prevention, Gay, Lesbian, Bisexual, Transgender, Two-Spirit, Queer, Canada, Suicide, prévention, hommes gais, femmes lesbiennes, personnes bisexuelles, personnes transgenres, queer, Canada

## Abstract

Suicide is a significant health issue among sexual and gender minority adults (SGMA); yet, there are no tailored suicide prevention programs for these marginalized populations in Canada. We hosted two world cafés with community leaders, health professionals, policymakers, and researchers to identify recommendations for mobilizing SGMA-focused suicide prevention programs. We identified five priorities: (1) make society safer for sexual and gender minorities; (2) decrease barriers to mental health services; (3) support community-driven and community-based interventions; (4) increase suicide knowledge and reduce stigma; (5) expand the knowledge base on SGMA suicide. In the absence of a national Canadian SGMA suicide prevention policy, these priorities provide a starting point in addressing SGMA suicide inequities by advancing SGMA-tailored interventions.

## Résumé

Le suicide est un problème de santé important chez les adultes issues des minorités sexuelles et de genre (AIMSG), par contre il n’existe aucun programme de prévention du suicide adapté à cette population au Canada. Nous avons donc organisé deux cafés du monde avec des dirigeants communautaires, des professionnels de la santé, des décideurs politiques et des chercheurs afin d’identifier des recommandations et des pistes de solutions au problème du suicide chez les AIMSG. Cinq priorités ont été identifiées : 1) rendre la société plus sécuritaire pour les AIMSG, 2) réduire les obstacles aux services en santé mentale, 3) soutenir les interventions et les efforts communautaires, 4) promouvoir les connaissances et réduire la stigmatisation liée au suicide chez les AIMSG, et 5) développer de nouvelles connaissances sur le suicide chez les AIMSG par la recherche. En l’absence d’une politique nationale canadienne de prévention du suicide pour les AIMSG, ces priorités fournissent un point de départ pour remédier aux inégalités relatives au suicide auxquelles font face les AIMSG et pour le développement d’interventions de prévention adaptées à cette population.

Sexual and gender minorities (SGM)—people identifying as lesbian, gay, bisexual, transgender, Two-Spirit, or queer—are at increased risk of suicide (Haas et al. 2011), but prevention interventions designed for this population are practically inexistent in Canada. This is due, in part, to the lack of knowledge related to specific risk factors of suicide among SGM, but also to the limited visibility of this population within the national suicide discourse to date (Government of Canada 2016; Haas et al. 2011). When SGM suicide is addressed, it has traditionally been treated as an issue affecting SGM youth (see https://www.canada.ca/en/public-health/services/suicide-prevention/suicide-canada.html)—despite a clearly established body of evidence indicating that the elevated risk of SGM suicide persists throughout SGM’s life course, including adulthood (Bauer et al. 2015; Blosnich et al. 2016). Increasingly, however, influential public health and policy groups (including Mental Health Commission of Canada, Canadian Mental Health Association) are acknowledging the high suicide risk of SGM adults (SGMA), yet few practicable solutions have been proposed to redress this inequity.

Meanwhile, other jurisdictions have brought together experts to identify the state of research and provide recommendations with regard to SGM suicide prevention (e.g., in the United States) (Haas et al. 2011), but these efforts have been conspicuously absent within Canada at the exception of efforts to mobilize around SGM youth suicide (Dyck 2012). Therefore, to stimulate suicide prevention efforts for Canadian SGMA, we gathered 49 stakeholders to participate in two world cafés to distill recommendations for suicide prevention for SGMA, generating five priorities and 20 recommendations (Table 1). The world cafés were held in Vancouver, British Columbia (October 2016 and November 2017), and included SGMA activists, front-line workers in mental health and/or SGMA health, policymakers, researchers, and people affected by SGMA suicide. Many spanned multiple roles, and the majority identified as members of SGMA communities. The world cafés consisted of a series of roundtable discussions that were self-facilitated around the question “What will it take to prevent suicide among SGMA?” with key points being recorded by note takers. Following the events, the notes were reviewed, double-coded, and summarized to identify recommendations for suicide prevention programs for SGMA.

**Table 1:** Priority actions for suicide prevention for sexual and gender minority adults (SGMA)

Priority area	Recommendations
1. Make society safer for sexual and gender minority adults	1. Support/build SGM-safe settings for adults
	2. Develop media guidelines on SGM-affirming reporting
	3. Reduce discrimination that exists within SGMA communities
	4. Respond to the Truth and Reconciliation Commission of Canada’s Calls to Action
2. Decrease barriers to mental health services	5. Increase the availability of free/low-cost, high-quality, culturally safe SGMA-affirming services
	6. Reduce wait times for existing free/low-cost SGMA-affirming services
	7. Clarify referral pathways for SGMA with suicidality
	8. Create SGMA-affirming practices and clinical spaces
	9. Explore new technologies to provide SGMA-affirming therapies to rural/remote SGMA
3. Support community-based interventions	10. Increase opportunities for SGMA to participate in social groups
	11. Provide suicide awareness training to existing SGMA social groups
	12. Create SGMA-specific suicide support groups
	13. Create a community suicide prevention grant program to sustain and scale interventions
4. Increase suicide knowledge and reduce suicide stigma	14. Provide suicide prevention training to SGMA
	15. Develop suicide literacy and suicide stigma campaigns
5. Expand the knowledge base on SGMA suicide	16. Identify SGMA-specific risk factors
	17. Explore the perspectives of under-researched SGMA groups and the connections between intersecting social identities and risk of suicide
	18. Monitor trends in SGMA suicide as a means to evaluating tailored interventions
	19. Increase the understanding of the lived experience of SGMA affected by suicide through qualitative research
	20. Develop knowledge using destigmatizing and empowering research approaches such as art-based and community-based methods

## Priority 1: Make society safer for sexual and gender minority adults

SGMA in Canada continue to experience violence and marginalization and these experiences increase the vulnerability of SGMA to suicide (Ferlatte et al. 2015). As such, making society safer for SGMA is a fundamental priority for suicide prevention. Specifically, GSAs (gender and sexuality alliances) and anti-homophobia/transphobia policies in schools are successful models of inclusion that promote safety which have been shown to reduce suicide among SGM youth (Saewyc et al. 2014). These models could be adapted to other contexts where SGMA experience discrimination (e.g., workplace and sports/recreation (Lloren and Parini 2017; Smith and Ingram 2004; Symons et al. 2017)), as they appear to contribute to supportive environments for SGM and have an impact on the social climate in which they are implemented. GSAs buffer against discrimination and other stressors (Saewyc et al. 2014); however, GSAs provide a benefit to current and future cohorts of SGM youth but do not benefit the majority of SGMA, who are no longer in school. More so, news media play an important role in shaping the general population’s views and attitudes towards SGM communities. However, media outlets often report on SGM issues in a way that is unintentionally stigmatizing. As such, SGM-affirming guidelines are needed for how SGM identities, experiences, and issues are represented by journalists, akin to journalistic guidelines for reporting suicides generally (Sinyor et al. 2018).

While SGMA communities can be a haven for some, many SGMA experience discrimination from *other* SGMA due to intersecting identities related to gender, sexuality, HIV status, race, Indigeneity, and class. Those who are marginalized within SGMA communities are often at increased risk of suicide (Ferlatte et al. 2018; Salway et al. 2018b). As such, actions to combat oppression within SGMA communities are urgently needed. First, culturally safe language guidelines are needed on social networking apps used by SGMA that forbid discriminatory languages common on these platforms (e.g., “no Asians”, “no fat”, “no fem”, “clean”), as these are spaces where discrimination is often unchecked. Second, SGMA community organizations and businesses should have clear anti-oppression policies (i.e., that prohibit racist, sexist, ableist, ageist, and classist behaviours and languages) posted in places that are visible and that have specific mechanisms for responding to discrimination. Third, non-Indigenous SGMA communities have a responsibility to respond to the Calls to Action of the Truth and Reconciliation Commission of Canada (Truth and Reconciliation Commission of Canada 2015), and to acknowledge and honour the experiences of Two-Spirit people (Indigenous SGMA). This is an important step towards strengthening the well-being of Indigenous SGMA as well as an important act of allyship to undertake in light of the high rates of suicide in Indigenous communities.

## Priority 2: Decrease barriers to mental health services

Removing mental health services barriers and increasing access to culturally safe services are critical steps to reducing suicide risk. First, structural changes are needed to the Canadian health care system to improve equitable access, including the implementation of publicly funded counselling services, which are currently difficult to access due to high out-of-pocket costs, as a Canadian SGMA survey recently identified inability to pay as the most prevalent barrier to mental health care access (Ferlatte et al. 2019c). While inability to afford mental health care is not unique to SGMA (Mesidor et al. 2011), SGMA are more likely to live in poverty (Kia et al. 2019; Pakula et al. 2016) and about half as likely to be partnered (Operario et al. 2015; Pakula et al. 2016) and as such may be less likely to access extended mental health coverage from a partner. This highlights the importance of extending mental health services coverage within the publicly funded health care system in Canada. More so, wait times need to be reduced for counselling services that are free or at low cost, and referral systems and protocols to access these services need to be simpler (i.e., self-referral) and provide quicker access to those struggling with suicidality. This could be achieved by adapting a combination of rapid same-day interventions and e-health services, which have been shown to be able to significantly reduce wait time (68% reduction) in the Canadian province of Newfoundland (Mental Health Commission of Canada 2019).

In addition, many mental health care providers are under-prepared to work with SGMA (Ferlatte et al. 2019a). Therefore, there is a need for evidence-based and comprehensive curricula for health professionals (including social workers, psychologists, nurses, and doctors) that specifically address the spectrum of suicide risk factors that SGMA experience. Specifically, health professionals need training to understand that there are multiple experiences within SGMA and to learn skills related to SGMA cultural safety and trauma-informed service delivery/practice to take into account traumatic experiences of SGMA-related discrimination, bullying, violence, and rejection, and their profound consequences on the mental health of SGMA (Baams et al. 2015; Hatzenbuehler 2009). A promising avenue is the expansion of mental health services within health services/agencies that are already trusted by SGMA and that are low barrier, such as services where individuals can self-refer and expect some degree of anonymity, as provided by some sexual health clinics (Salway et al. 2018a). Finally, new technologies (such as text messaging, chat options) could play an important role in facilitating access to mental health support to rural SGMA communities, a group that is particularly underserved.

## Priority 3: Support community-based interventions

Community-based interventions that address the social determinants of health are critical to suicide prevention. First, interventions to support social connectedness are needed, such as social groups. Formalized social groups already exist for SGMA but they could more effectively address suicide prevention by training group leaders to recognize signs of suicide and develop the competencies to connect suicidal individuals to interventions. There is also a need for support groups for SGMA struggling with suicidality. Suicidal SGMA could benefit from hearing others share their stories about suicide (Ferlatte et al. 2019a) and how they managed their suicidal thoughts while breaking their isolation. Given that both SGMA status and suicide are stigmatized, the development of safer spaces to facilitate these groups is especially important. Further evaluation of these interventions will be critical to determine whether SGMA-focused are more effective than universal interventions. Finally, a community grants program should be implemented for individuals to start and evaluate their own prevention initiatives, which may tap into the talent, resourcefulness, and skills of SGMA communities.

## Priority 4: Increase suicide knowledge and reduce suicide stigma

Stigma surrounding suicide, compounded by poor knowledge of suicide risks, can prevent people with thoughts of suicide from seeking help (Sudak et al. 2008). In the case of SGMA, this stigma may be compounded by stigma related to SGMA status. SGMA are not always knowledgeable of suicide risk factors, of the common signs of suicidality, and of the options for supportive services. As such, there is a need for interventions in SGMA communities to increase mental health literacy and to destigmatize suicide.

To increase suicide literacy and reduce suicide stigma among SGMA, peers should be trained in suicide prevention given that literature shows that peers (such as friends) are a common source of emotional support in times of crisis (Rickwood et al. 2007). A recent Canadian survey has found that SGMA are largely interested in learning skills related to suicide and have a strong preference for online learning (Ferlatte et al. 2019b). As such, an online training that takes into account the specific context and risk factors of SGMA should be developed to assist SGMA in identifying and intervening with suicidal SGMA peers. In addition, an increase in messaging about mental health within SGMA spaces is needed, including social marketing efforts focused on suicide that adapt some of the successful efforts in social marketing that have been used in the realm of SGMA sexual health promotion (Vega and Roland 2005). Such campaigns could inform SGMA about suicide prevention services available and challenge misinformation to help destigmatize suicide. There is also a need for accessible online suicide resources that provide information on suicide risk factors and treatment options that are tailored to the specific experiences and needs of SGMA. Finally, social media represent an underexplored and untapped—yet important—opportunity to promote suicide literacy.

## Priority 5: Expand the knowledge base on SGMA suicide

Multiple knowledge gaps related to SGMA suicide continue to challenge the development of suicide prevention programs (Haas et al. 2011). Therefore, a long-term approach to sustainable research with regard to SGMA suicide prevention is required. First, detailed trend analyses are essential to identify potential inequities and improvement in suicide rates among diverse subgroups of the SGMA population. These trend analyses will require that, in addition to the current-standard SGMA variables (Bauer et al. 2017), suicidality variables be included as core content in federally funded surveys, such as the Canadian Community Health Survey. Second, an equity-based approach to research is proposed to investigate suicide inequities and specific risk factors across multiple social axes within SGMA communities, such as race, Indigeneity, class, gender, and geography.

Suicide research has been dominated by quantitative analyses (Hjelmeland and Knizek 2010) and accordingly researchers should diversify their approaches. Specifically, qualitative research is needed to provide nuanced understandings of suicidality among SGMA. Additionally, building on the success of a recent Canadian study using photography (Ferlatte et al. 2019a), arts-based methodologies are proposed to engage SGMA in destigmatizing and empowering research approaches. Finally, community-based research is critical to ensure that research findings are relevant and receive wide community support. SGMA with lived experience of suicidality must be meaningfully included in the research process and be compensated for their collaboration.

## Conclusion

We consulted experts to generate a detailed list of priority areas and recommendations to respond to the unique contemporary contexts and circumstances of SGMA suicide in Canada. In the absence of a formal strategy, we suggest that these recommendations can be used to generate new interventions, policies, and research projects. Beyond these recommendations, the processes and recommendations drawn from our consultation reveal innovation and strong commitment to addressing longstanding social issues that contribute to suicide among SGMA. Our challenge is to harness these energies to collectively build, sustain, and scale interventions that will reverse the suicide inequity experienced by this population. We conclude that it is now time to mobilize suicide prevention for SGMA in Canada.
